# Comparison of serum acylcarnitine levels in patients with myalgic encephalomyelitis/chronic fatigue syndrome and healthy controls: a systematic review and meta-analysis

**DOI:** 10.1186/s12967-023-04226-z

**Published:** 2023-06-19

**Authors:** Ryuhei Jinushi, Sakue Masuda, Yuki Tanisaka, Sho Nishiguchi, Kento Shionoya, Ryo Sato, Kei Sugimoto, Takahiro Shin, Rie Shiomi, Akashi Fujita, Masafumi Mizuide, Shomei Ryozawa

**Affiliations:** 1 Department of Gastroenterology, Saitama Medical University International Medical Center, 1397-1 Yamane, Hidaka, Saitama 350-1298 Japan; 2 Department of Gastroenterology Medicine Center, Shonan Kamakura General Hospital, 1370-1 Okamoto, Kamakura, Kanagawa, 247-8533 Japan; 3 Department of General Internal Medicine, Shonan Kamakura General Hospital, 1370-1 Okamoto, Kamakura, Kanagawa, 247-8533 Japan; 4 Graduate School of Medicine, International University of Health and Welfare, 4-1-26 Akasaka, Minato-ku, Tokyo, 107-8402 Japan

**Keywords:** Acylcarnitine, Chronic fatigue syndrome, COVID-19, Myalgic encephalomyelitis, Systemic exertion intolerance disease

## Abstract

**Background:**

Myalgic encephalomyelitis/chronic fatigue syndrome/systemic exertion intolerance disease (ME/CFS/SEID) is a condition diagnosed primarily based on clinical symptoms, including prolonged fatigue and post-exertional malaise; however, there is no specific test for the disease. Additionally, diagnosis can be challenging since healthcare professionals may lack sufficient knowledge about the disease. Prior studies have shown that patients with ME/CFS/SEID have low serum acylcarnitine levels, which may serve as a surrogate test for patients suspected of having this disease. This systematic review and meta-analysis aimed to investigate the differences in serum acylcarnitine levels between patients with ME/CFS/SEID and healthy controls.

**Methods:**

This systematic review was conducted using PubMed and Ichushi-Web databases. Following the Preferred Reporting Items for Systematic Reviews and Meta-Analyses statement, we included all studies from the databases’ inception until February 17, 2023, that evaluated blood tests in both patients with ME/CFS/SEID and healthy control groups. The primary endpoint was the difference in serum acylcarnitine levels between the two groups.

**Results:**

The electronic search identified 276 studies. Among them, seven met the eligibility criteria. The serum acylcarnitine levels were analyzed in 403 patients with ME/CFS/SEID. The patient group had significantly lower serum acylcarnitine levels when compared with the control group, and the statistical heterogeneity was high.

**Conclusion:**

The patient group had significantly lower serum acylcarnitine levels when compared with the control group. In the future, the measurement of serum acylcarnitine levels, in addition to clinical symptoms, may prove to be a valuable diagnostic tool for this condition.

**Supplementary Information:**

The online version contains supplementary material available at 10.1186/s12967-023-04226-z.

## Background

Myalgic encephalomyelitis/chronic fatigue syndrome (ME/CFS) is a chronic disease characterized by a broad range of symptoms, including central nervous system abnormalities, gastrointestinal dysfunction, morbid fatigue, muscle pain, and cognitive impairment that does not improve with adequate rest [1]. Despite extensive research, the pathogenesis of ME/CFS is not yet understood, and there are no specific tests or biological markers for its diagnosis [2]. Currently, the Centers for Disease Control-1994/Fukuda criteria are often used to diagnose ME/CFS [3]. However, approximately 80% of ME/CFS cases are not correctly diagnosed. Patients with ME/CFS are often not diagnosed with ME/CFS and are referred to medical facilities with indefinite complaints [4]. Therefore, it takes longer to identify patients with ME/CFS and their symptoms worsen [5].

Despite some existing research on ME/CFS, many healthcare professionals may still lack awareness of the disease [6]. However, the coronavirus disease 2019 (COVID-19) pandemic, caused by severe acute respiratory syndrome coronavirus-2 (SARS-CoV-2) has brought greater attention to ME/CFS [7]. Typical acute phase symptoms of COVID-19 include fever, cough, and smell and taste disorders [8]. Notably, many patients experience COVID-19 sequelae long after they have recovered from the peak of the disease than from the acute phase symptoms of COVID-19. Typical sequelae such as post-exertional malaise (PEM), unrefreshing sleep, and orthostatic intolerance are considered the clinical symptoms of ME/CFS [5]. In 2015, the National Academy of Medicine developed diagnostic criteria for ME/CFS and proposed a new umbrella term, systemic exertion intolerance disease (SEID), to replace ME/CFS [5, 9]. The current diagnostic criteria for ME/CFS/SEID are based solely on symptoms. However, the diagnosis of the disease will become less challenging as more specific blood tests and imaging findings for the disease become available in the future.

In this study, we collected and reviewed articles in which serum acylcarnitine, free carnitine, and total carnitine levels were measured in patients with ME/CFS/SEID. In our previous case report on post-COVID-19 ME/CFS/SEID, we speculated that viral infection may have adversely affected energy metabolism in muscular tissue, resulting in decreased serum acylcarnitine levels [10]. Against this background, the measurement of serum acylcarnitine levels may be useful for the diagnosis of ME/CFS/SEID. Therefore, we identified studies that included blood tests for patients diagnosed with ME/CFS/SEID. Next, we analyzed serum acylcarnitine, free carnitine, and total carnitine levels in patients with ME/CFS/SEID and in healthy controls. Finally, we conducted a systematic review and meta-analysis to evaluate their usefulness for future ME/CFS/SEID diagnosis.

## Methods

### Protocol registration

This systematic review and meta-analysis was conducted in compliance with the Preferred Reporting Items for Systematic Reviews and Meta-Analyses statement (Additional file 1: Table S1) [11]. Our study protocol was registered in the University Hospital Medical Information Network Clinical Trials Registry (UMIN-CTR) on March 1, 2023 (UMIN-CTR: UMIN000050465; URL: https://center6.umin.ac.jp/cgi-open-bin/ctr_e/ctr_view.cgi?recptno=R000057475).

### Eligibility criteria

The inclusion criteria for our systematic review were as follows: (1) systematic reviews and prospective and retrospective studies of patients with ME/CFS/SEID, (2) articles published in Japanese or English, and (3) articles searched using PubMed (MEDLINE) and Ichushi-Web (NPO Japan Medical Abstracts Society) on February 17, 2023.

The exclusion criteria for our systematic review were as follows: (1) guidelines, narrative reviews, case reports, and conference presentations on ME/CFS/SEID, (2) studies that did not measure serum acylcarnitine, free carnitine, or serum total carnitine levels, and (3) articles published in languages other than Japanese or English.

### Literature search strategy

This systematic review was conducted using the databases PubMed and Ichushi-Web. The search included articles available from the inception of the databases up to February 17, 2023. The following electronic search terms were used to retrieve the literature in PubMed: (“Carnitine Acyltransferases”[MeSH] OR “acylcarnitine”[tiab] OR “COVID-19”[tiab] OR “COVID-19”[MeSH] OR “COVID-19 Vaccines”[tiab] OR “COVID-19 Vaccines”[MeSH] OR “COVID-19 serotherapy”[tiab] OR “COVID-19 serotherapy”[tiab] OR “COVID-19 Nucleic Acid Testing”[tiab] OR “covid-19 nucleic acid testing”[MeSH] OR “COVID-19 Serological Testing”[tiab] OR “covid-19 serological testing”[MeSH] OR “COVID-19 Testing”[tiab] OR “covid-19 testing”[MeSH] OR “SARS-CoV-2”[tiab] OR “sars-cov-2”[MeSH] OR “Severe Acute Respiratory Syndrome Coronavirus 2”[tiab] OR "NCOV”[tiab] OR “2019 NCOV”[tiab]) AND (“fatigue syndrome, chronic”[MeSH] OR chronic fatigue syndrome[tiab] OR myalgic encephalomyelitis[tiab] OR systemic exertion intolerance disease[tiab]). In addition, an electronic search strategy was created on Ichushi-Web based on the aforementioned search terms.

### Literature selection

**Fig. 1:**
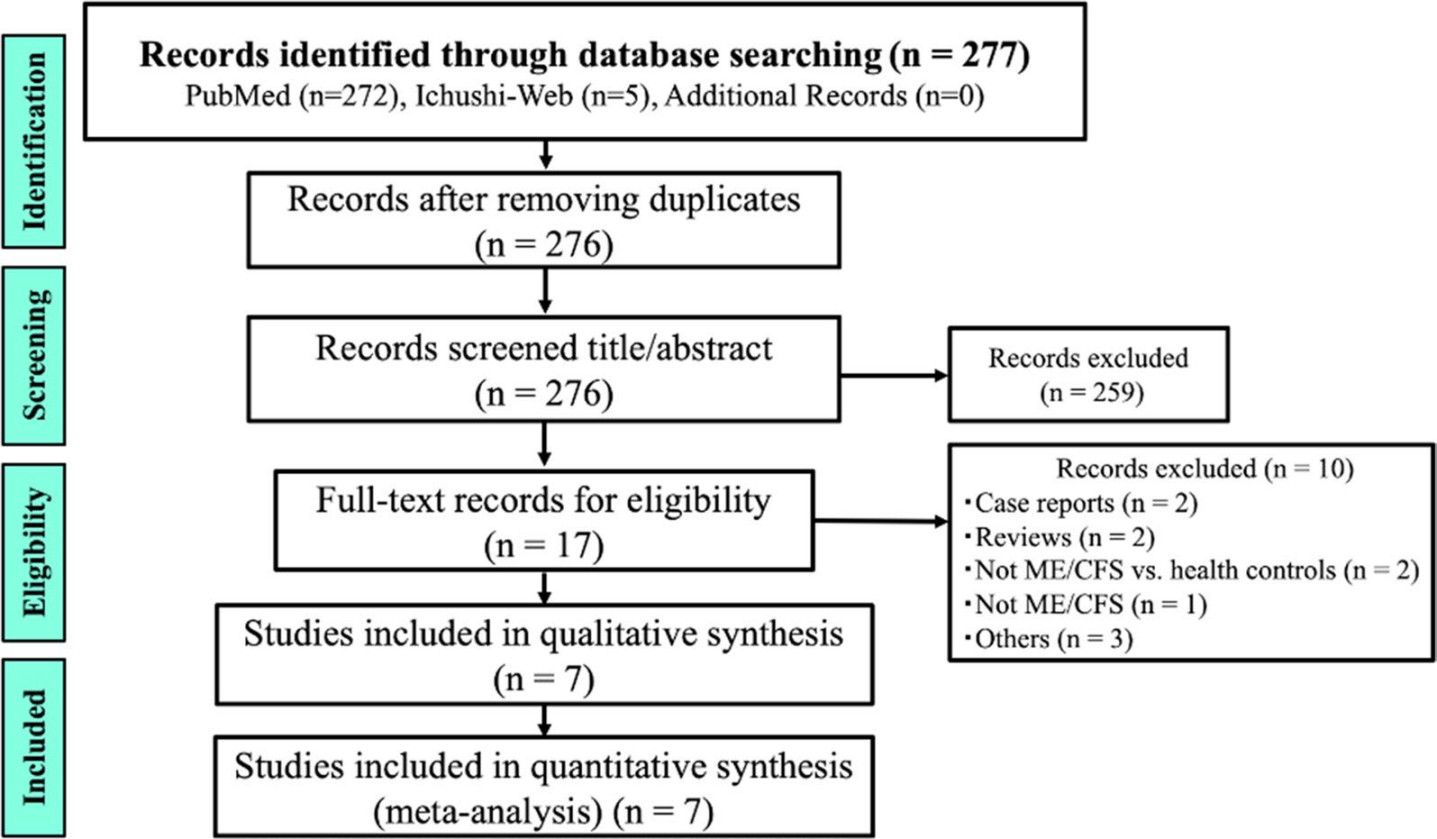
The PRISMA flow diagram for the selection of studies (searched on February 17, 2023). *PRISMA* Preferred Reporting Items for Systematic Reviews and Meta-Analyses

Figure 1 presents a flowchart of the literature collection process. First, we searched the literature on COVID-19, ME/CFS/SEID, and serum acylcarnitine levels, resulting in 272 studies from PubMed and five studies from Ichushi-Web. Out of the 276 articles initially identified, 259 were excluded during primary screening due to unrelated titles and abstracts to ME/CFS/SEID, and one duplicate article was removed. Next, 17 studies were subjected to secondary screening, and a systematic review was conducted on nine datasets extracted from seven studies [12–18]. None of the studies were extracted from other sources.

### Data extraction and quality assessment

A data extraction Excel sheet was created and included the following data: study design, title, first author, publication journal, publication year, number of patients with ME/CFS/SEID, sex, age, and blood test findings such as serum total carnitine levels, free carnitine levels, and acylcarnitine levels. The Newcastle–Ottawa Scale (NOS) was used for quality assessment (Additional file 2: Table S2).

### The ME/CFS/SEID diagnostic criteria

The following ME/CFS/SEID diagnostic criteria were used in the seven studies included in this systematic review: the diagnostic criteria proposed by Holmes et al. [19] Kitani et al. [20] Fukuda et al. [3] and the Working Group of the Royal Australasian College of Physicians [21].

### Definition of primary and secondary endpoints

The primary endpoint was the difference in serum acylcarnitine levels between the patients with ME/CFS/SEID and healthy controls. As secondary endpoints, differences in serum total and free carnitine levels were examined.

### Data synthesis and statistical analysis

We compiled an Excel sheet with the number of participants in each study, the mean and standard deviation (SD) of serum acylcarnitine levels of each study’s patient group, and the mean and SD of the serum acylcarnitine levels of each study’s control group. The differences in serum acylcarnitine levels between the two groups were calculated, and forest plots were generated based on a random-effects model to account for interstudy bias. The results were expressed as 95% confidence intervals (CI) for the difference in serum acylcarnitine levels between the two groups, and the statistical heterogeneity was assessed by Breslow (τ^2^), Higgins (I^2^), and Birge’s ratio (H2) indices. The I^2^ statistics were classified as < 30%, 30–60%, 61–75%, and > 75% with low, moderate, and high heterogeneity, respectively. In our study, the H2 statistics were classified as < 40%, 30–60%, 50–90%, and 75–100%, and were defined as low heterogeneity, moderate heterogeneity, possibly high heterogeneity, and high heterogeneity, respectively. Publication bias was tested by examining funnel plot. Statistical significance was set at p < 0.05. All analyses were performed using STATA^®^ version 17 software (StataCorp, College Station, TX, USA).

## Results

### Characteristics of this systematic review

The initial search identified 276 studies, excluding one duplicate. Following screening against our inclusion and exclusion criteria, 17 studies remained. Of these, seven were ultimately included in the meta-analysis [12–18]. Among the seven studies included, one [15] provided information on both Swedish and Japanese patients. Data from Japanese and Swedish patients are reported separately (Table 1). These studies involved a total of 403 patients with ME/CFS/SEID and 893 healthy controls, and were published between 1992 and 2010, with most of them being conducted in Japan. In addition, all seven studies were retrospective in nature. The mean overall quality score on the NOS for the included studies was 6.9 (SD, 0.4) (Additional file 2: Table S2).

**Table 1:** Characteristics of the seven studies included in this meta-analysis

Study	Journal	Study design	Sample size, n^a^	Sex female, n (%)	Age (SD or range)	ME/CFS/SEID patient group, n	Healthy control group, n
Kuratsune H, et al. [12]	Nihon rinsho	Retrospective study	59	34 (57.6)	35.9 (14–62)	27	41
Kuratsune H, et al. [13]	Clinical infectious diseases	Retrospective study	38	19 (50)	No data	38	308
Plioplys AV, et al. [14]	Neuropsychobiology	Retrospective study	35	27 (77.1)	40 (16–67)	M 8, F 27	M 40, F 45
Kuratsune H, et al. [15]^b^	International journal of molecular medicine	Retrospective study	57	57 (100)	No data	57	46
Kuratsune H, et al. [15]^c^	International journal of molecular medicine	Retrospective study	146	82 (56.2)	No data	M 64, F 82	M 177, F 131
Soetekouw PM, et al. [16]	The Netherlands journal of medicine	Retrospective study	25	25 (100)	35.9 (9.8)	25	25
Jones MG, et al. [17]	Clinica chimica acta	Retrospective study	31	19 (61.3)	M 42 (26–63), F 42 (21–84)	31	31
Reuter SE, et al. [18]	Journal of internal medicine	Retrospective study	44	27 (61.4)	49.9 (15)	44	49

^a^The number of patients diagnosed with ME/CFS/SEID according to the diagnostic criteria [3, 19–21]

^b^Characteristics of Swedish patients with ME/CFS/SEID [15]

^c^Characteristics of Japanese patients with ME/CFS/SEID [15]

*ME* myalgic encephalomyelitis, *CFS* chronic fatigue syndrome, *SEID* systemic exertion intolerance disease, *n* number, *SD* standard deviation, *M* male, *F* female

### Outcomes of differences in serum carnitine levels between the ME/CFS/SEID patient group and the control group

**Fig. 2:**
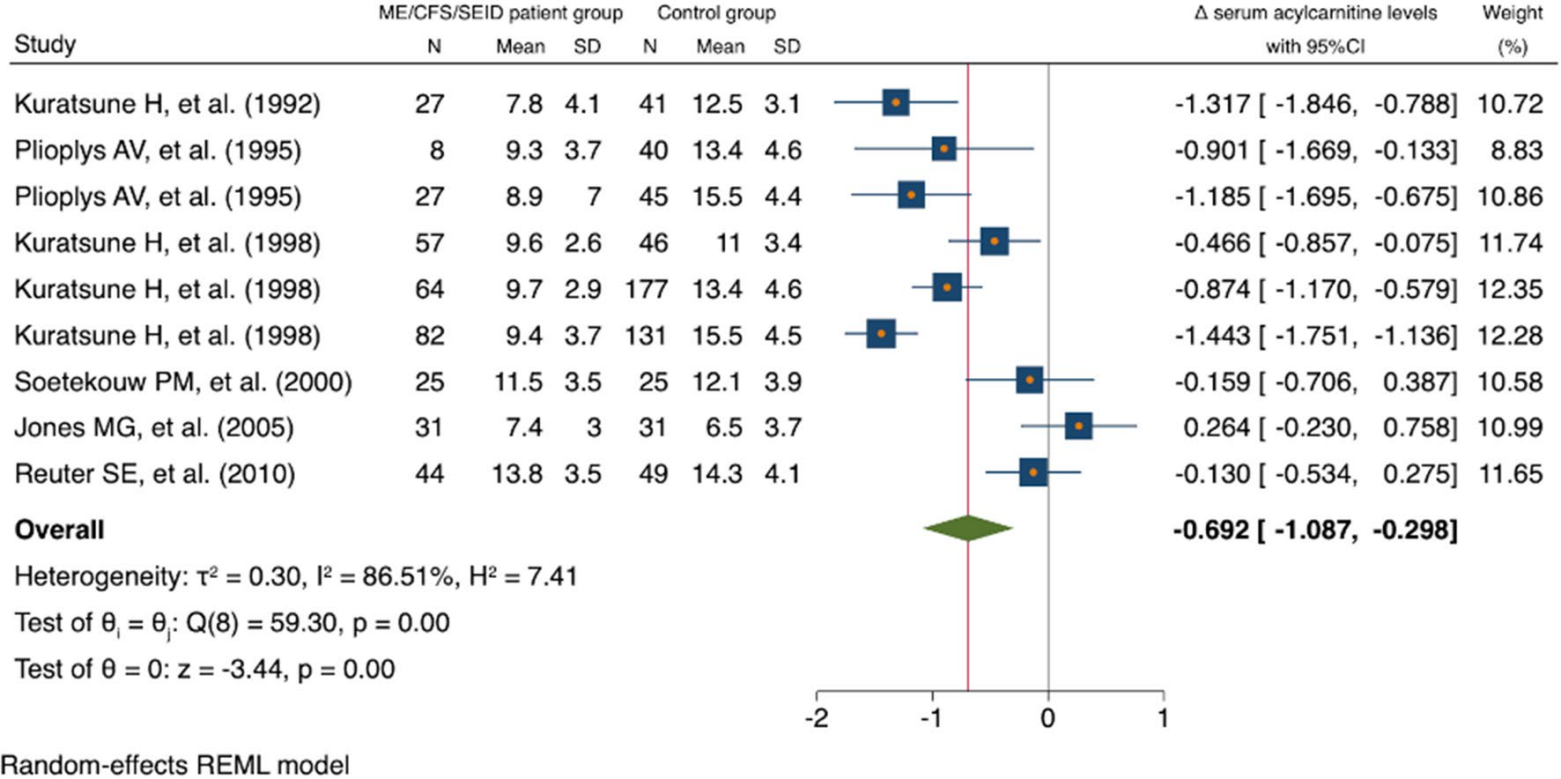
Forest plot for serum acylcarnitine levels between the ME/CFS/SEID patient group and the control group. The ME/CFS/SEID patient group had significantly lower serum acylcarnitine levels when compared with the control group (Δ = − 0.69; [95% CI − 1.09, − 0.30]), with a high heterogeneity. *ME* myalgic encephalomyelitis, *CFS* chronic fatigue syndrome, *SEID* systemic exertion intolerance disease, *CI* confidence interval, *N* number, *SD* standard deviation

In our study, serum acylcarnitine levels were obtained from 403 patients with ME/CFS/SEID. Among them, we compared the serum acylcarnitine levels of 365 of these patients with those of healthy controls. Table 2 shows the serum total carnitine levels and free carnitine levels between the ME/CFS/SEID patient group and the control group. Nine serum total carnitine and free carnitine datasets were collected from the seven studies included in the meta-analysis [12–18]. Although some values were missing, the majority of studies did not demonstrate a significant difference in serum total carnitine and free carnitine levels between the two groups. As presented in Table 3, serum acylcarnitine levels from the ME/CFS/SEID patient group and the control group were collected from 11 datasets from the seven studies included in the meta-analysis [12–18]. Two of these datasets had missing values, and forest plots were created from the remaining nine datasets. Based on the analysis of these nine datasets, our study found that the ME/CFS/SEID patient group had significantly lower serum acylcarnitine levels when compared with the healthy control group (Δ = − 0.69; [95% CI − 1.09, − 0.30]), with a high statistical heterogeneity of 86.5% (Fig. 2).

**Table 2:** Results of serum total carnitine levels and free carnitine levels between the ME/CFS/SEID patient group and the healthy control group

	Total carnitine levels, μmol/L (SD)			Free carnitine levels, μmol/L (SD)		
Study	ME/CFS/SEID patient group	Healthy control group	P-value^a^	ME/CFS/SEID patient group	Healthy control group	P-value^a^
Kuratsune H, et al. [12]	No data	No data		51.8 (9.5)	56.2 (9.7)	NS
Kuratsune H, et al. [13]	No data	No data		M 53.5 (8.5), F 46.8 (8.2)	No data	NS
Plioplys AV, et al. [14]	M 49.9 (9.1), F 41.2 (9.5)	M 59.3 (11.9), F 51.5 (11.6)	p < 0.05	M 40.6 (8.9), F 32.1 (6.9)	M 46.8 (10.0), F 40.1 (9.5)	p < 0.05
Kuratsune H, et al. [15]^b^	No data	No data		34.8 (7.8)	38.5 (8.1)	p < 0.05
Kuratsune H, et al. [15]^c^	No data	No data		No data	M 56.1 (10.7), F 43.6 (10.0)	
Soetekouw PM, et al. [16]	46.9 (7.6)	48.9 (8.2)	NS	35.4 (7.6)	36.8 (6.5)	NS
Jones MG, et al. [17]	40.5 (8.7)	43.1 (9.8)	NS	33.2 (7.9)	36.6 (9.5)	NS
Reuter SE, et al. [18]	58.8 (13.6)	59.5 (12.9)	NS	45.0 (11.3)	45.2 (9.79)	NS

^a^In each study, those for which a p-value was not calculated are shaded

^b^The data in Swedish [15]

^c^The data in Japanese [15]

*ME* myalgic encephalomyelitis, *CFS* chronic fatigue syndrome, *SEID* systemic exertion intolerance disease, *SD* standard deviation, *M* male, *F* female, *NS* not significant

**Table 3:** Results of serum acylcarnitine levels between the ME/CFS/SEID patient group and the healthy control group

	Acylcarnitine levels, μmol/L (SD)		
Study	ME/CFS/SEID patient group	Healthy control group	P-value
Kuratsune H, et al. [12]	7.8 (4.1)	12.5 (3.1)	p < 0.001
Kuratsune H, et al. [13]^a^	M 8.7 (3.3)	No data	p < 0.001
Kuratsune H, et al. [13]^a^	F 8.4 (4.4)	No data	p < 0.001
Plioplys AV, et al. [14]^b^	M 9.3 (3.7)	M 13.4 (4.6)	p < 0.00001
Plioplys AV, et al. [14]^b^	F 8.9 (7.0)	F 15.5 (4.4)	p < 0.00001
Kuratsune H, et al. [15]^c^	9.6 (2.6)	11.0 (3.4)	p < 0.001
Kuratsune H, et al. [15]^d^	M 9.7 (2.9)	M 13.4 (4.6)	p < 0.001
Kuratsune H, et al. [15]^d^	F 9.4 (3.7)	F 15.5 (4.5)	p < 0.001
Soetekouw PM, et al. [16]	11.5 (3.5)	12.1 (3.9)	NS
Jones MG, et al. [17]	7.4 (3.0)	6.5 (3.7)	NS
Reuter SE, et al. [18]	13.8 (3.5)	14.3 (4.1)	NS

^a^The data in the upper section are for males and the data in the lower section are for females [13]

^b^The data in the upper section are for males and the data in the lower section are for females [14]

^c^The data in Swedish [15]

^d^The data in the upper section are for Japanese males and the data in the lower section are for Japanese females [15]

*ME* myalgic encephalomyelitis, *CFS* chronic fatigue syndrome, *SEID* systemic exertion intolerance disease, *SD* standard deviation, *M* male, *F* female, *NS* not significant

### Publication bias

**Fig. 3:**
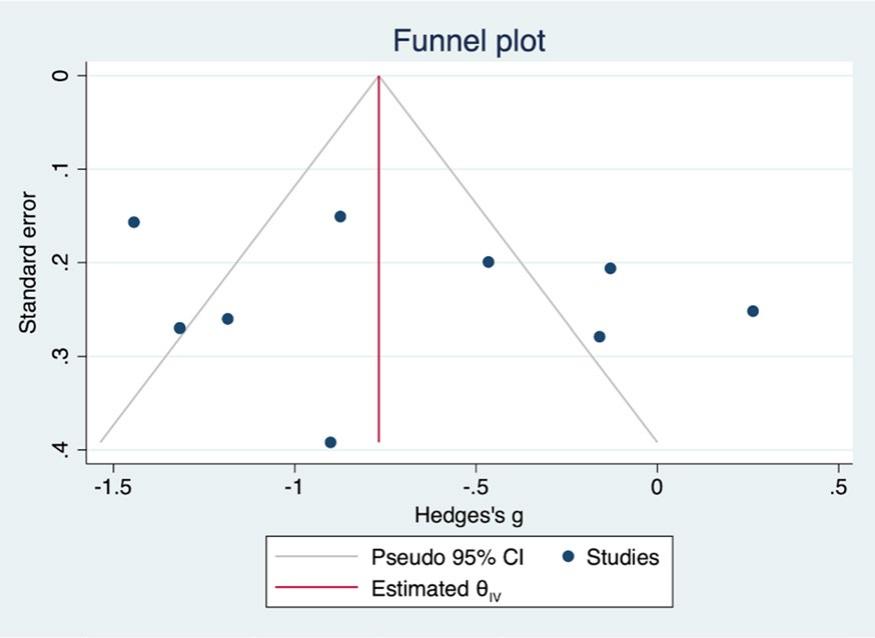
Funnel plot for serum acylcarnitine levels between the ME/CFS/SEID patient group and the control group. No publication bias was visually determined in this funnel plot. *ME* myalgic encephalomyelitis, *CFS* chronic fatigue syndrome, *SEID* systemic exertion intolerance disease.

No publication bias was found from the funnel plot of studies comparing serum acylcarnitine levels in the ME/CFS/SEID patient group and healthy control group (Fig. 3).

## Discussion

CFS is a syndrome characterized by chronic unexplained fatigue and muscular pain; however, its cause remains unknown. In 1988, Holmes et al. proposed CFS as the new name for chronic Epstein–Barr virus syndrome, [19] which led to further studies of the disease by Kitani et al. [20] Fukuda et al. [3] and the Working Group of the Royal Australasian College of Physicians [21]. However, its diagnosis is difficult because of the variety of clinical symptoms and the lack of specific tests. Moreover, healthcare professionals often have limited knowledge of the disease, as the pathophysiology of ME/CFS/SEID is not typically taught thoroughly in medical education. This lack of knowledge contributes to the low diagnosis rate, with only approximately 20% of patients with this disease being correctly diagnosed [4, 22].

In recent years, this disease has been increasingly reported as a sequela of COVID-19 and is gradually being recognized [8]. We have previously reported a case of the disease after recovery from COVID-19 [10]. In addition to respiratory symptoms, COVID-19 sequelae present with a variety of endocrine and metabolic disorders such as thyroid dysfunction and diabetes mellitus, gastrointestinal symptoms such as abdominal pain and diarrhea, skin symptoms such as alopecia and dermatitis, and extreme muscular fatigue and general malaise related to the disease [23].

In the course of researching mental health problems post-COVID-19, we realized that many patients have psychiatric disorders such as depression and anxiety due to incorrect diagnosis and treatment of ME/CFS/SEID, which led us to this systematic review, in the hope of helping to solve these problems. The cause of this disease has been suggested to be a type of viral infection, and the diagnostic criteria specify unexplained fatigue for more than 6 months, PEM, and unrefreshing sleep as core symptoms [8]. In 2015, the National Academy of Medicine proposed SEID as the new name to replace ME/CFS [5].

After a thorough review of the literature on ME, CFS, and SEID reported to date, we hypothesize that low serum acylcarnitine levels in patients with ME/CFS/SEID may be due to an impaired mitochondrial fatty acid oxidation cascade caused by viral infections, including COVID-19 [24, 25]. Normally, in the mitochondrial matrix, fatty acids taken up by cells undergo β-oxidation to produce ATP. In addition, long-chain fatty acids are converted to acyl-CoA in cells, which reacts with carnitine to produce acylcarnitine [26]. Carnitine is an essential factor in the aforementioned cascade.

Kuratsune et al. studied acylcarnitine and carnitine levels in patients with ME/CFS/SEID. The results showed that acylcarnitine levels were lower in patients with ME/CFS/SEID and that lower acylcarnitine levels may correlate with fatigue levels. It was then considered that patients with ME/CFS/SEID may exhibit symptoms such as general malaise and fatigue due to decreased brain uptake of acylcarnitine and consequently decreased synthesis of neurotransmitters such as gamma-aminobutyric acid and glutamate [12–15, 25].

We encountered a case in which we were able to accurately diagnose this disease based on literature [10]. At present, the relationship between ME/CFS/SEID and serum acylcarnitine levels remains unclear; however, we hope that the measurement of serum acylcarnitine levels will be useful in the future. Measurement of serum acylcarnitine levels is a minimally invasive and reliable test that can be widely used without radiation exposure or high costs. Based on our systematic review and meta-analysis, ME/CFS/SEID should be suspected in patients presenting with subjective symptoms of long-term unexplained chronic fatigue, PEM, unrefreshing sleep, cognitive impairment, and orthostatic intolerance. Measurement of serum acylcarnitine levels may assist in the diagnosis.

This study had some limitations. First, the ME/CFS/SEID diagnostic criteria were not standardized in each study [3, 19–21]. Second, the sample size of each study was limited, and no studies related to this topic has been published in recent years. Third, there was some bias in the nationalities, sex, and body size of the patients with ME/CFS/SEID in the seven retrospective studies included in this meta-analysis. Finally, the method used to measure serum acylcarnitine levels differed slightly between the studies.

Overall, our systematic review and meta-analysis suggest the utility of measuring serum acylcarnitine levels in patients with ME/CFS/SEID. Further studies comparing and examining laboratory findings, including serum acylcarnitine levels, in patients with ME/CFS/SEID and in healthy controls may contribute to more patients being correctly diagnosed with ME/CFS/SEID.

## Conclusion

In cases where ME/CFS/SEID is suspected based on clinical symptoms, the measurement of serum acylcarnitine levels may contribute to a more definitive diagnosis of the disease. In addition, the blood test is highly versatile with extremely low invasiveness and may become more widely used in the future.

## Supplementary Information

**Additional file 1: Table S1.** PRISMA 2020 statement

**Additional file 2: Table S2.** NOS for assessing the quality of retrospective studies included in the systematic review

## Data Availability

The data that support the findings of this study are available from the corresponding authors (RJ) upon reasonable request.

## Abbreviations

ME: Myalgic encephalomyelitis
CFS: Chronic fatigue syndrome
PEM: Post-exertional malaise
SEID: Systemic exertion intolerance disease
PRISMA: Preferred reporting items for systematic reviews and meta-analyses
COVID-19: Coronavirus disease 2019
SARS-CoV-2: Severe acute respiratory syndrome coronavirus-2
NOS: Newcastle–ottawa scale
SD: Standard deviation
CI: Confidence interval
