# Supplementary material for: Comparison of serum acylcarnitine levels in patients with myalgic encephalomyelitis/chronic fatigue syndrome and healthy controls: a systematic review and meta-analysis
Source: J Transl Med. 2023 Jun 19;21:398. doi: 10.1186/s12967-023-04226-z (PMC10280864; doi:10.1186/s12967-023-04226-z)
Supplement: Supplementary file 2 — Additional file 2: Table S2. NOS for assessing the quality of retrospective studies included in the systematic review [file 12967_2023_4226_MOESM2_ESM.docx]

**Additional file 2: Table S2**

**NOS for assessing the quality of retrospective studies included in the systematic review.**

| **Study** | **Selection** | **Comparability** | **Exposure** |
| --- | --- | --- | --- |
| Kuratsune H, et al.^12^ (1992) | ★★★ | ★ | ★★ |
| Kuratsune H, et al.^13^ (1994) | ★★★ | ★ | ★★★ |
| Plioplys AV, et al.^14^ (1995) | ★★★ | ★ | ★★★ |
| Kuratsune H, et al.^15^ (1998) | ★★★ | ★ | ★★★ |
| Soetekouw PM, et al.^16^ (2000) | ★★★ | ★ | ★★★ |
| Jones MG, et al.^17^ (2005) | ★★★ | ★ | ★★★ |
| Reuter SE, et al.^18^ (2010) | ★★★ | ★ | ★★★ |
